# The COVID-19 Pandemic and Dental Professionals’ Infection Risk Perception: An International Survey

**DOI:** 10.3390/jcm12216762

**Published:** 2023-10-26

**Authors:** Guglielmo Campus, Magdalena Marie-Luise Jenni, Marcela Diaz Betancourt, Maria Grazia Cagetti, Rodrigo A. Giacaman, David J. Manton, Gail V. A. Douglas, Joana C. Carvalho, Thomas Gerhard Wolf

**Affiliations:** 1 Department of Restorative, Preventive and Pediatric Dentistry, School of Dental Medicine, University of Bern, 3010 Bern, Switzerland; 2 Department of Surgery, Microsurgery and Medicine Sciences, School of Dentistry, University of Sassari, Viale San Pietro, 07100 Sassari, Italy; 3 Department of Biomedical, Surgical and Dental Science, University of Milan, 20142 Milan, Italy; 4 Cariology and Gerodontology Units, Department of Oral Rehabilitation, Faculty of Dentistry, University of Talca, Talca 3460000, Chile; 5 Cariology, Centrum voor Tandheelkunde en Mondzorgkunde, Universitair Medisch Centrum Groningen, University of Groningen, 9713 AV Groningen, The Netherlands; 6 Department of Dental Public Health, School of Dentistry, University of Leeds, Leeds LS2 9JT, UK; 7 Faculty of Medicine and Dentistry, UCLouvain, 1200 Brussels, Belgium; 8 Department of Periodontology and Operative Dentistry, University Medical Center of the Johannes Gutenberg University Mainz, 55131 Mainz, Germany

**Keywords:** COVID-19, dentistry, dental professionals, global health, infection control, infection risk, oral health, public health, SARS-CoV-2

## Abstract

A global survey among dentists was used to identify the various impacts of the COVID-19 pandemic on this professional group. Special attention was given to perception and assessment of infection risk. From May to August 2020, the questionnaire was delivered in 36 countries by respective research groups and was completed by 52,491 dental professionals. The survey was designed as a cross-sectional survey based on a previously standardized questionnaire. This study focuses on the part of the questionnaire that deals with the perception of the infection risk of COVID-19 by dentists and their patients. A logistic regression model was used, which consisted of four Likert items as response options and the additional self-reported routine or emergency treatment as the dependent variable. Analysis by continent found that European and Asian dentists were particularly likely to be infected at work (OR = 1.45 95%CI = 1.02/1.84 and OR = 2.68, 95%CI = 1.45/3.22, respectively), while it was likely that Australian dentists did not feel particularly at risk due to low infection rates. Three quarters of Americans treated only emergencies during this survey period, while Europeans (64.71%) and Asians (66.67%) provided mostly routine care. This could affect the Europeans’ confidence that they would not be able to protect themselves from infections in the long-term. The COVID-19 pandemic and its impact on dental professionals’ infection risk perception is determined by the geographical origin of dentists. This study shows that, especially in high-incidence countries, infection risk perception was higher when dentists tried to provide routine dental procedures to their patients. Dental professionals can offer themselves and their patients good protection by maintaining high standards of hygiene. However, their concerns should be taken seriously and the dental professionals’ group that is of great importance for oral health care and prevention, should not be neglected in the future, even in the event of emerging pandemics.

## 1. Introduction

It has been just over 3 years since SARS-CoV-2 (severe acute respiratory syndrome from coronavirus type 2) plunged the world into a pandemic. As of 15 September 2023, 695,289,957 cases of virus infection and more than 6.9 million deaths have been reported worldwide [1]. USA and India are the countries with the highest number of infections [1]. Thanks to continued vaccination worldwide, the next waves of infection are not expected to be as aggressive as in the past three years [2]. Still, airborne droplets or direct contact with contaminated material, surfaces, or infected cases remains the reason for the human-to-human transmission of the SARS-CoV-2 [3]. Therefore, in addition to vaccination, it is recommended to avoid close contact and to remain in quarantine in the presence of respiratory symptoms [4].

Due to the high rate of virus transmission through the respiratory system, dentists and dental clinics have received close attention in the past three years [5,6,7]. Dental personnel are known to work in the oral cavity with potentially high viral and bacterial exposure [7]. Due to the high droplet and aerosol procedures, dental healthcare personnel are in the highest risk category and in the front-line for exposure to SARS-CoV-2 [7]. The anxiety of dentists increases not only because of the risk of infection, but also because of isolation, economic changes, and job stress [8,9]. During the first months of the pandemic, dentists worldwide were asked to limit procedures to emergency dental care and emergencies only, precisely because they were considered to be a group of healthcare professionals at very high risk of infection [10]. However, the behavior of dentists during the pandemic has not been homogenous. In some countries, dental care was limited, while in other countries non-emergency care was also provided due to local or national legal restrictions [11,12,13].

A global survey among dental professionals has been developed and performed with the aim of evaluating the impact of the SARS-CoV-2/COVID-19 pandemic in different countries around the world through a large multicenter study [14]. Due to extensive data collected from this global study, this is the second paper on dental professionals and the SARS-CoV-2/COVID-19 pandemic. The aim of this paper by the COVIDental collaboration group was to report on infection risk perception of dental professionals in 36 countries on five continents.

## 2. Materials and Methods

### 2.1. Development and Questionnaire

The international survey was created, as previously described, with a modified Delphi method during the first wave of the SARS-CoV-2/COVID-19 pandemic between April and August 2020 [14,15]. Using the Stehr–Green scale, the questionnaire was structured into three domains: (1) personal data including age, gender, area of living and working, and working status; (2) dental professionals’ infection rate and symptoms/signs presumably related to the COVID-19; and (3) working conditions and personal protective equipment (PPE) adopted after the outbreak of the infection [14]. The originally English questionnaire is presented in the Supplementary Materials (Table S1) [15]. The Italian survey was used as a pretest and explained in previous studies [15,16]. Twelve dental professionals participated in a pre-test, achieving an intraclass correlation coefficient (ICC) of at least 0.80 for each item. As this is a formal and global study, it follows the CHERRIES guidelines. The study protocol has been previously described and registered in the World Pandemic Research Network (WPRN) with number WPRN-486352 [16]. Thirty-six research groups around the world were set up to collaborate on the survey. Each team was instructed and informed by a central coordinator (G.C.), who coordinated a central management team (M.G.C., M.D.B., T.G.W.) [14]. The original Italian version of the questionnaire was first translated into English and then into the local languages of the respective research groups by a member of each group. The researcher had to recognize the conceptual equivalence in his or her language and have an excellent command of English. A further requirement for the researcher was specialist knowledge of public health dentistry. All participants received the same core questionnaire, a description of the intent of the study, an online informed consent (according to individual country data protection laws) and, in addition, supplementary questions from each national team (optional) [14]. Finally, a back translation from the different language into English was carried out by a translator, who was not part of the research team [16].

### 2.2. Participants and Study Size

To proceed with the study, the number of active dental professionals in each country that participated was collected. To reach the target of 5% participants, invitations to participate were requested at a proportion of 5–20% in each country. Each country team was able to use the strategies and modalities best suited to their country to reach the largest number of dentists in both public and private health care systems. Direct mailings to individual dental professionals were used, as well as invitations to participate through national dental associations and other related organizations and social media outreach [14].

### 2.3. Bias Control and Ethics Concern

The platform that conducted the survey and collected the data was designed to avoid duplicate responses. The project protocol and the consent were approved by the local Ethics Committee in some countries, if required. If the online informed consent was not accepted/signed by a participant, the questionnaire was not evaluated and excluded. Each applicant was able to review their answers, also, all participants received a unique identification number, based on their IP address. A Research Electronic Data Capture (REDCap; Vanderbilt University, Nashville, TN, USA) account provided a secure repository, where all data were stored [14]. REDCap is a secure web application for creating and managing online surveys and databases (www.project-redcap.org; accessed on 23 October 2023).

### 2.4. Independent Variables and Data Sources

As independent variables, there were several data entered and updated by the national research teams, i.e., limited clinical activities to emergency only, the gross national income (GNI) per capita 2019 and purchasing power parity (PPP, international dollar), and the community positive rate (CPR) during the survey period. This has been described in a previously published article [14].

### 2.5. Outcome Variables

The variables analyzed in the present paper related to the dental professionals’ perception that COVID-19 infection represented a risk both for themselves during work and for the patients treated. Both items were completed with a Likert scale with four possible options (Unlikely, Very unlikely, Likely, Very likely).

### 2.6. Statistical Analysis

All data collected were entered into an Microsoft Excel spreadsheet (Version 16.77, Microsoft Corporation, Redmond, WA, USA) and quality-checked for correctness. STATA16™ (Version 17, StataCorp LLC, College Station, TX, USA) was used for statistical analysis. The analysis was performed by the management team (G.C., M.M.L.J., M.D.B., M.G.C., T.G.W.) at the central level. No demographic data were missing and absolute as well as relative frequencies were calculated for each item.

Countries were grouped according to continents (Europe, Africa, Asia, Americas, and Oceania). If a cell had a value of less than five, differences in proportions were evaluated with the Chi-square test or the Fisher exact test. Multiple testing for post-hoc estimation, such as the number of observed frequencies, expected frequencies, percentage, and contribution to the Chi-square were run. Estimation of a nonparametric test for trends across the areas with different prevalence of COVID-19 self-reported rates and questionnaire items were also calculated. The effect size was calculated using the Cramer’s V, as a measure of the strength of association among the levels of the row and column variables. Logistic regression analysis was run using dental professionals’ perception that COVID-19 is a risk for both dentists and patients; dentists’ awareness of avoiding COVID-19 infection at work as a dependent variable.

## 3. Results

### 3.1. Personal Data

**Figure 1:**
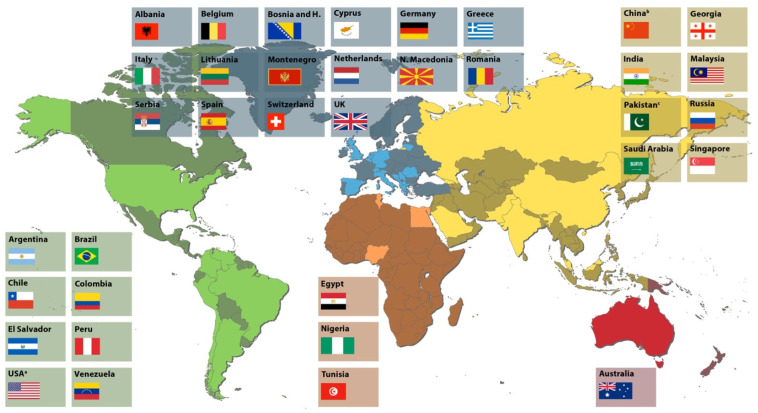
Participating countries are divided into continents (green: North and South America; blue: Europe; orange: Africa; yellow, Asia; red: Oceania). ^a^ USA/California; ^b^ China/Hubei; ^c^ Pakistan/Lahore.

A total of 52,491 dental professionals from 36 countries completed the questionnaires. Participating countries are shown in Figure 1. The gender ratio of dentists in most countries favored women. Switzerland had the highest percentage of male respondents, while Russia had 100% female participation. Dentists from Nigeria, China (Hubei Province), Malaysia, Singapore, and the United Kingdom most often reported working in the national healthcare system, in universities or as administrative staff. More than 50% of the dentists from countries such as Argentina, Peru, USA/California, Venezuela, Australia, Albania, Belgium, Cyprus, Chile, Germany, Greece, Italy, Lithuania, Macedonia, Montenegro, Netherlands, Romania, Spain, and Switzerland were owners of a private practice. Most respondents were general dentists (43.2% vs. 23.61% specialists), but a third of the participants did not answer this item.

### 3.2. COVID-19 as a Risk for Dentists

In Europe and Asia, dentists considered the COVID-19 infection risk for both themselves and their patients very likely (Table 1). American dentists were almost significantly similar, whether they saw a risk of COVID-19 infection for themselves and their patients or not. Only in Africa and Oceania (Australia) split opinions arose on the risk of infection.

### 3.3. COVID-19 as a Risk for Patients

Table 2 shows the opinions of dentists on the risk of COVID-19 infection for patients in different countries, the positivity rate at the national level and of dentists working in each country, and the sex ratio.

### 3.4. Risk for Patients throughout Routine or Emergency Services

Table 3 shows dental professionals’ opinions on the risk of COVID-19 infection for patients in the different continents and the respective percentage of dentists who performed routine or emergency treatments. The results show that in Asia and Europe, most dentists (66.67% and 64.71%, respectively) continued to provide routine dental treatment. In these same continents, dentists perceived a higher infection risk for patients (Europe 39.65% and Asia 46.89%). However, in Africa, where all dentists continued to provide routine dental treatment, the infection risk for patients was judged very likely by only 32.45% of responders. Finally, in America, where three quarters of dentists (75.00%) provided only emergency treatment during the questionnaire period, the percentage of ‘Very likely’ (32.59%) was similar to that on the African continent.

### 3.5. Awareness of Infection

Descriptive analysis of Table 4 shows that mostly European dentists were not confident about avoiding infection with SARS-CoV-2/COVID-19 at work. America and Asia were divided, on one side participants seemed confident of not becoming infected, Asia more so, on the other hand there were dental workers who were not confident about avoiding the infection. Another factor of awareness is the use of protective equipment, for example eye protection and FFP2/N95 masks; this was analyzed in the first paper of the study [14]. Africa, on the other hand, was confident, and Oceania was the most confident continent regarding avoiding infection (Table 4).

## 4. Discussion

This paper deals with awareness and perception of risk infection of dental professionals during the COVID-19 pandemic worldwide. Since dentists are considered front-line workers in the health care system and are exposed to infection through aerosols and close patient contact, it is important to place this professional group and to take their concerns and fears seriously. The results of this paper show that dentists around the world consider COVID-19 infection risk to be high both for themselves and their patients, especially among those who continued to provide routine dental treatment during the first wave of the infection. However, it was difficult to make a comparison between the different countries and continents, as the study took place at different times of the respective infectious events within each country. Therefore, the study is certainly only a snapshot of the time [17,18,19]. In Australia, for example, there was a nationwide lockdown with severe entry restrictions at the time of the survey, which could explain the lower fear of infection among dental practitioners [20]. The Australian government’s high level of surveillance and low number of cases may have helped dentists avoid fear of contamination [20]. Dentists from African countries, on the other hand, have not clearly decided whether there was a risk for themselves when treating patients. It is important to consider that many African regions have conducted fewer tests and may have been misinformed about the risk of COVID-19 [21,22,23]. While European dentists were not confident about avoiding infection with SARS-CoV-2/COVID-19 at work, the high numbers of infections at that time in Europe and the media presence on the topic were most likely decisive for the anxiety and awareness of the infection. However, differences between the respective continents may have multiple causes such as treating emergencies and reducing the number of patient appointments.

A central aspect regarding the limitations of this research project is the composition of the present sample. Despite the high number of respondents, it is not possible to generalize the results as, for example, Europe is represented by many more countries than Africa, where only Egypt, Nigeria and Tunisia answered, and Oceania is represented only from Australia. Previous studies show that among health care workers, the fear of infection was very high (71%) and the fear of infecting others was much higher (85%) [24]. The results of the current paper may help colleagues understand that high hygiene standards certainly provide protection against the infection, as the culture of effective infection control in the dental profession has proven for decades. However, the results of this survey should also be a wake-up call to politicians so that the dental profession is not left alone with their concerns and fears. In addition to the fear of infection, the warning by the WHO and the media not to undergo dental treatment because of risk of infection has also led to financial problems for dentists [12,25].

The period of data collection was not homogeneous across the different countries and especially with different phases of the pandemic and this could explain the high variability of results. When dental offices are closed or routine treatments are canceled, dentists can also experience depression and fear of failure; because business viability is at risk due to a lack of patients [9,26]. Furthermore, it can be assumed that patients do not receive regular treatment, and certain diseases such as oral cancer or aggressive pathologies are detected later than hoped. In Australia, the government has clearly stood up for the people, closing the borders to protect them and as a consequence, Australian dentists show that they care little about being infected. In countries where fewer tests were performed, dentists appear to be less frightened, but a greater risk for the population is possible. In fact, high-income countries were found to have a very high number of infections, but a low number of deaths, while low-income countries had a high number of deaths with low infection rates [27]. One Health cooperation, which has been discussed for years, could therefore potentially be a way in the future to act globally and jointly and to reduce the gap between low-income countries and high-income countries and to fight future pandemics together [28,29].

Even though the present study was conducted early in the pandemic, it is currently possible that new variants of the virus and new waves of infection will be unleashed, but with milder symptoms and provision of better protection through vaccination. Nevertheless, the dental practice and clinic remains a place where the risk of infection is always possible, and dentists must therefore adhere to national and international guidelines for infection prevention. For this reason, protective measures such as eye protection, FFP2/N95 masks, and regular ventilation of rooms and use of rinsing solutions before treating patients remain essential precautions, according to international documents, that dentists should be aware of. In addition, there are already informative international brochures and papers for the dental profession to read and utilize [30,31,32]. Particularly, overworked dental professionals with previous illnesses and fear of infecting themselves and others, are at risk of experiencing psychological impairments, which again influence their perception [33]. High standards of hygiene and effective information and guidelines can provide effective protection for dentists worldwide; however, the fears, perceptions, and concerns of dental professionals in relation to the risk of infection must be taken seriously [34].

## 5. Conclusions

The COVID-19 pandemic and its impact on dentists in terms of perception and infection risk are different worldwide and are mainly determined by the geographical origin of dentists. In countries where the incidence was high, risk assessment and perception of potential infection were higher when dentists tried to maintain regular practice and offered routine treatments to their patients, as shown by results from Europe and Asia. Dentists in countries such as Australia where infection rates were low at the time of the survey, also had a lower perception of the risk of infection.

Our aim was to assess the impact of the COVID-19 pandemic in different countries worldwide, in a comprehensive multicenter study. The differences between continents and regions may have been influenced by the chronology of regional outbreaks and the rapidity of response to COVID-19. The results reflect socioeconomic and cultural differences and inequalities in different countries that are also reflected in the dental care system. The perception of infection risk also increases psychological distress, and this can lead to subjective overload, which can have many consequences in the healthcare system and its workers.

## Figures and Tables

**Table 1:** Logistic regression using dentists’ perception that SARS-CoV-2/COVID-19 was a risk for the dentist as a dependent variable.

	Very Unlikely	Unlikely	Likely	Very Likely
	OR 95%CI	OR 95%CI	OR 95%CI	OR 95%CI
**Africa**	1.04 (0.57/0.84)	1.23 (1.07/1.24)	1.35 (0.99/1.53)	1.28 (0.86/1.54)
**Americas**	1.20 (0.91/1.49)	0.93 (0.72/0.94)	1.25 (1.14/1.50)	2.00 (0.95/3.40)
**Asia**	1.02 (0.66/1.27)	1.42 (1.04/1.73)	1.22 (1.02/1.53)	2.68 (1.45/3.22)
**Europe**	0.87 (0.63/1.01)	1.06 (0.84/1.31)	1.45 (1.02/1.84)	2.01 (1.32/2.95)
**Oceania**	0.82 (0.58/0.85)	0.82 (0.62/0.94)	1.25 (1.03/1.51)	1.74 (1.06/2.74)

**Table 2:** COVID-19 positive rate at the country level (global and for dentists) and risk for the patient as perceived by dental professionals. ^a^ USA/California; ^b^ China/Hubei; ^c^ Pakistan/Lahore, ^ data calculated by the authors.

	Risk for the Patient						
Country	Positive Rate SARS-CoV-2/ COVID-19 %	Dentists Positive Rate	Dentist M/F Ratio	Very Unlikely	Unlikely	Likely	Very Likely
		%		*n* (%)	*n* (%)	*n* (%)	*n* (%)
**Albania**	39.14	0.00	0.52	24 (15.79)	26 (17.11)	21 (13.82)	81 (53.29)
**Argentina**	34.43	2.41	0.30	103 (11.68)	123 (13.95)	219 (24.83)	437 (49.55)
**Australia**	0.69	0.61	0.63	156 (13.40)	281 (24.14)	186 (15.98)	541 (46.48)
**Belgium**	2.68	2.35	0.73	271 (33.54)	111 (13.74)	184 (22.77)	242 (29.95)
**Bosnia**	17.0	1.88	0.43	59 (27.70)	35 (16.43)	35 (16.43)	84 (39.44)
**Brazil**	30.15	5.02	0.25	726 (39.78)	305 (16.71)	580 (31.78)	214 (11.73)
**Chile**	10.98	2.12	0.49	245 (10.60)	415(17.95)	718 (31.06)	934 (40.40)
**China ^b^**	0.00	0.72	0.55	431 (33.18)	475 (36.57)	209 (16.09)	184 (14.16)
**Colombia**	26.60	6.46	0.38	675 (15.60)	526 (12.16)	1862 (43.03)	1264 (29.21)
**Cyprus ^^^**	0.49	0.00	0.71	57 (17.01)	33 (9.85)	61 (18.21)	184 (54.93)
**Egypt ^^^**	24.41	3.09	1.31	131 (14.46)	230 (25.39)	324 (35.76)	221 (24.39)
**El Salvador**	12.40	1.47	0.50	86 (15.84)	58 (10.68)	162 (29.83)	237 (43.65)
**Georgia ^^^**	1.48	1.26	0.38	46 (14.51)	36 (11.36)	95 (29.97)	140 (44.16)
**Germany**	0.70	0.69	1.43	833 (31.36)	522 (19.65)	476 (17.92)	825 (31.06)
**Greece**	0.44	0.00	0.80	7 (15.22)	4 (8.70)	14 (30.43)	21 (45.65)
**India**	10.92	1.81	0.86	416 (12.60)	284 (8.60)	422 (12.78)	2180 (66.02)
**Italy**	2.90	1.12	0.66	1386 (16.73)	1340 (16.18)	2705 (32.65)	2853 (34.44)
**Lithuania**	0.46	0.49	0.13	0 (0.00)	36 (18.75)	48 (25.00)	108 (56.25)
**Macedonia**	10.4	0.00	0.60	--	--	6 (25.00)	18 (75.00)
**Malaysia**	0.09	0.39	0.13	490 (16.87)	459 (15.81)	504 (17.36)	1451 (49.97)
**Montenegro ^^^**	14.84	0.00	1.11	31 (28.18)	13 (11.82)	25 (22.73)	41 (37.27)
**Netherlands**	1.00	1.90	0.47	74 (20.11)	80 (21.74)	88 (23.91)	126 (34.24)
**Nigeria**	2.10	0.00	1.62	56 (13.49)	41 (9.88)	68 (16.39)	250 (60.24)
**Pakistan ^c^**	16.27	17.83	0.40	127 (31.05)	86 (21.03)	93 (22.74)	103 (25.18)
**Peru**	23.55	3.19	0.60	498 (23.97)	334 (16.07)	404 (19.44)	842 (40.52)
**Romania**	4.72	0.76	0.26	105 (9.99)	214 (20.36)	462 (43.96)	270 (25.69)
**Russia**	12.55	14.68	0.00	14 (1.41)	224 (22.56)	230 (23.16)	525 (52.87)
**Saudi Arabia**	5.17	7.84	0.86	321 (38.31)	150 (17.90)	179 (21.36)	188 (22.43)
**Serbia**	4.06	0.00	0.35	75 (5.14)	341 (23.36)	346 (23.70)	698 (47.81)
**Singapore**	7.30	0.84	0.41	--	--	43 (42.57)	58 (57.43)
**Spain**	4.06	3.19	0.53	711 (30.67)	359 (15.49)	340 (14.67)	908 (39.17)
**Switzerland**	1.26	0.91	1.97	403 (30.44)	563 (42.52)	144 (10.88)	214 (16.16)
**Tunisia**	1.51	4.13	0.52	354 (44.31)	102 (12.77)	126 (15.77)	217 (27.16)
**UK**	1.09	1.08	0.85	581 (13.50)	145 (3.37)	1007 (23.40)	2571 (59.74)
**USA ^a^**	7.00	0.89	0.63	189 (33.63)	140 (24.91)	115 (20.46)	118 (21.00)
**Venezuela ^^^**	5.46	0.00	0.26	111 (14.64)	201 (26.52)	162 (21.37)	284 (37.47)
***p*-value**				<0.01	<0.01	<0.01	<0.01

**Table 3:** Dental professionals’ opinions on the COVID-19 infection risk for the patients in the different continents and the respective percentage of dentists who performed routine or emergency treatments during the first wave of the pandemic.

	Type of Treatments		Very Unlikely	Unlikely	Likely	Very Likely	Total
	Emergency *n* (%) *	Routine *n* (%) *	*n* (%)	*n* (%)	*n* (%)	*n* (%)	*n* (%)
**Africa**	--	3 (100.00)	541 (25.52)	373 (17.59)	518 (24.43)	688 (32.45)	2120 (4.21)
**Americas**	6 (75.00)	2 (25.00)	2633 (19.82)	2102 (15.82)	4222 (31.77)	4330 (32.59)	13,287 (26.37)
**Asia**	3 (33.33)	6 (66.67)	1987 (19.23)	1771 (17.14)	1731 (16.75)	4845 (46.89)	10,334 (20.51)
**Europe**	6 (35.29)	11 (64.71)	4631 (18.80)	4046 (16.42)	6192 (25.13)	9769 (39.65)	24,638 (48.91)
***p*-value**	<0.01		0.01	0.08	<0.01	<0.01	--

* Percentage expressed by row.

**Table 4:** Logistic regression using dentists’ awareness of avoiding COVID-19 infection at work as the dependent variable.

	Not Confident	Slightly Confident	Fairly Confident	Confident
	OR 95%CI	OR 95%CI	OR 95%CI	OR 95%CI
**Africa**	1.14 (1.00/1.66)	0.83 (0.67/1.08)	1.40 (1.06/1.64)	1.12 (0.96/1.33)
**Americas**	1.43 (1.10/1.74)	0.93 (0.68/0.92)	1.14 (0.94/1.47)	1.27 (1.01/1.55)
**Asia**	1.57 (1.20/1.85)	1.18 (1.02/1.60)	1.28 (0.93/1.53)	1.72 (1.10/2.82)
**Europe**	1.87 (1.09/2.72)	1.34 (1.02/1.41)	0.97 (0.73/1.04)	0.93 (0.75/1.06)
**Oceania**	1.32 (0.99/1.59)	0.82 (0.56/0.89)	1.05 (0.83/1.43)	1.64 (1.11/2.36)

## Data Availability

The data presented in this study are available on request from the corresponding author. The data are not publicly available due to data protection reasons.

## Supplementary Materials

The following supporting information can be downloaded at: https://www.mdpi.com/article/10.3390/jcm12216762/s1, Table S1: English questionnaire.
