# Supplementary material for: The COVID-19 Pandemic and Dental Professionals’ Infection Risk Perception: An International Survey
Source: J Clin Med. 2023 Oct 26;12(21):6762. doi: 10.3390/jcm12216762 (PMC10650638; doi:10.3390/jcm12216762)
Supplement: Supplementary file 1 [file jcm-12-06762-s001.zip › jcm-2621239-supplementary.pdf]

Table S1.S Questionnaire

Gender<br><br>Age<br>Zip Code (living)<br>Zip Code (working)	male<br>female	
Working status	Private dentist<br>Private/NHS<br>NSH	
From the start of the COVID-19 you had	No symptoms<br>You resulted COVID-19 positive<br>You were hospitalized for COVID<br>I had one/more symptoms	Fever<br>Cough<br>Fatigue<br>Short Breath<br>Nasal congestion<br>Headache<br>Rhinorrhea<br>Sore throat<br>Diffuse pain<br>Diarrhea<br>Anosmia<br>Ageusia<br>Conjunctivitis
Only if you work in the NHS, are you currently working?	Yes<br>No	
From the 21st February	You kept working as usual<br>You limited your activity to emergencies<br>You have stopped all activities	
If you have limited the activity to emergencies, when did you start limiting?	Between February 21-23<br>Between 24 February and 1 March<br>Between March 2-6<br>Between March 7-14<br>After March the 14th	
If you have stopped the activity, when it is happened?	Between February 21-23<br>Between 24 February and 1 March<br>Between March 2-6<br>Between March 7-14<br>After March the 14th	
In the case that you have continued working after February 21st, which of the following precautionary measures have you taken?	None<br>Phone Triage<br>Appointments reduced so as not to saturate the waiting room<br>Postponement of therapies in elderly patients, or with systemic diseases	

	Handle disinfection several times a day<br>Disinfection of push buttons, POS, chairs, several times a day<br>Verify the patient's current health status on access<br>Detecting the patient's body temperature<br>Detection of body temperature of all co-workers and leave of those with a temperature above 37.5°.<br>Washing the patient's hands<br>Space of at least one meter between patients<br>Mask for the patient<br>Frequent ventilation of waiting rooms<br>Removal of magazines and books from the waiting area<br>Storage of coats, bags and other items outside the operating area<br>Pre-operative rinse with mouthwash containing 1% hydrogen peroxide<br>Pre-operative rinse with mouthwash containing chlorhexidine 0.12-0.2%<br>Pre-operative rinse with mouthwash containing 0.2-1% iodopovidone<br>Pre-operative rinse with mouthwash containing alcohol and essential oils<br>Pre-operative rinse with mouthwash containing Cetylpyridinium chloride at 0.05-0.10%<br>Rinse with diluted mouthwash<br>Ventilation of the operating area for at least 10 minutes after each patient<br>Surface disinfection with 70% ethyl alcohol<br>Disinfection of surfaces with 0.5% sodium hypochlorite<br>Usual disinfectant with others active ingredients<br>Washing operators' hands before and after each procedure<br>Removal of all disposable protective devices and disinfection of non-disposable devices
Which of the following protections have you adopted?	Surgical mask<br>FFP2 or FFP3 facial filters<br>Disposable headset<br>Sterile microfiber disposable gown<br>Water-repellent TNT disposable gown<br>Disposable gown<br>Safety glasses or visor<br>Sterile disposable gloves<br>Disposable gloves<br>Rotating instrument with anti-retraction valve
Did you follow a course on Covid-19?	Yes<br>No
Do you think that you know enough on COVID?	Yes<br>No
	Unlikely<br>Very unlikely

Do you believe that the infection by SARS-CoV-2 is a risk for the dentist?	Likely<br>Very likely
Do you believe that the infection by SARS-CoV-2 is a risk for the dentist?	Unlikely<br>Very unlikely<br>Likely<br>Very likely
How sure are you that you can avoid becoming infected with SARS-CoV-2 during work?	No confident<br>Enough confident<br>A bit confident<br>Confident
Do you believe that in a health emergency situation such as the current one, the risk of infection transmission in the dental practice is	Less than the risk runs going in a supermarket<br>Comparable to the risk runs going in a supermarket<br>Higher than the risk runs going in a supermarket
